# Multimodal imaging of spontaneous subretinal hemorrhage in a young male: a case report

**DOI:** 10.1186/s12886-020-01634-3

**Published:** 2020-09-22

**Authors:** He-he Hu, Xiao-yu Zhu, Zheng-gao Xie, Fang Chen

**Affiliations:** 1 Department of Ophthalmology, Northern Jiangsu People’s Hospital Affiliated to Yangzhou University, Yangzhou, China; 2 Department of Ophthalmology, Xijing Hospital of the Air Force Medical University, Xi’an, China; 3 Department of Ophthalmology, Affiliated Hospital of Yangzhou University, Yangzhou, China; 4 Department of Ophthalmology, Gulou Hospital Affiliated to Medical College of Nanjing University, Nanjing, China

**Keywords:** Spontaneous subretinal hemorrhage, Hypertension, Multimodal imaging

## Abstract

**Background:**

Spontaneous subretinal hemorrhage (SSRH) is a rare disease that severely affects the visual function, and is difficult to diagnose. This study aimed to describe the multimodality imaging characteristics of SSRH in a young male patient.

**Case presentation:**

A 28-year-old male was presented to our hospital with “sudden drop of left eye vision for one week.” Three weeks ago, he was admitted to other hospital due to sudden severe pain and unclear vision in the left eye for 1 h. The intraocular pressure was 69 mmHg, and the blood pressure was 230/120 mmHg. Skull CT and MRI detected abnormal signal shadows in the left eye and no abnormalities in the brain. B-ultrasonography indicated occupying lesions in the left eye. Two weeks later, the patient came to our hospital for treatment as the vision of the left eye had decreased sharply. Admission examination: blood pressure was 200/120 mmHg, best-corrected visual acuity was 20/20 in the right eye and hand motion in the left eye. Fundus details could not be evaluated in the left eye because of hemorrhage in the vitreous cavity. B-ultrasonography of the left eye revealed a dense, diffuse intravitreal hemorrhage. Skull MRI showed an abnormal signal shadow in the left eyeball, suggesting intraocular hemorrhage. Vitrectomy revealed a dome-shaped lesion in the peripheral part of the inferotemporal region during the operation. Postoperative indocyanine green angiography (ICGA) of the lesion showed hypofluorescence and no leakage or altered morphology during the whole imaging process. Follow-up showed gradual reabsorption of SSRH.

**Conclusions:**

In this case, SSRH was considered to be associated with high blood pressure. Multimodal imaging provides accurate data for the diagnosis and follow-up of the disease.

## Background

SSRH is a rare disease that is often difficult to diagnose and severely affects the visual function. It could be easily misdiagnosed as choroidal melanoma especially when it occurs in the peripheral retina with vitreous hemorrhage, which will be managed by enucleation. Multimodal imaging plays a vital role in the diagnosis of this disease. All cases of SSRH reported in the literature have many predisposing factors, including anticoagulant therapy, cachexia, or hypertension [1]; however, that caused by a single factor of hypertension has not yet been reported.

This case aimed to improve the clinician’s understanding of SSRH based on the characteristics of multimodal imaging. Thus, the application and analysis of imaging diagnosis of the disease need an in-depth investigation to reduce misdiagnosis and mistreatment.

## Case presentation

**Fig. 1:**
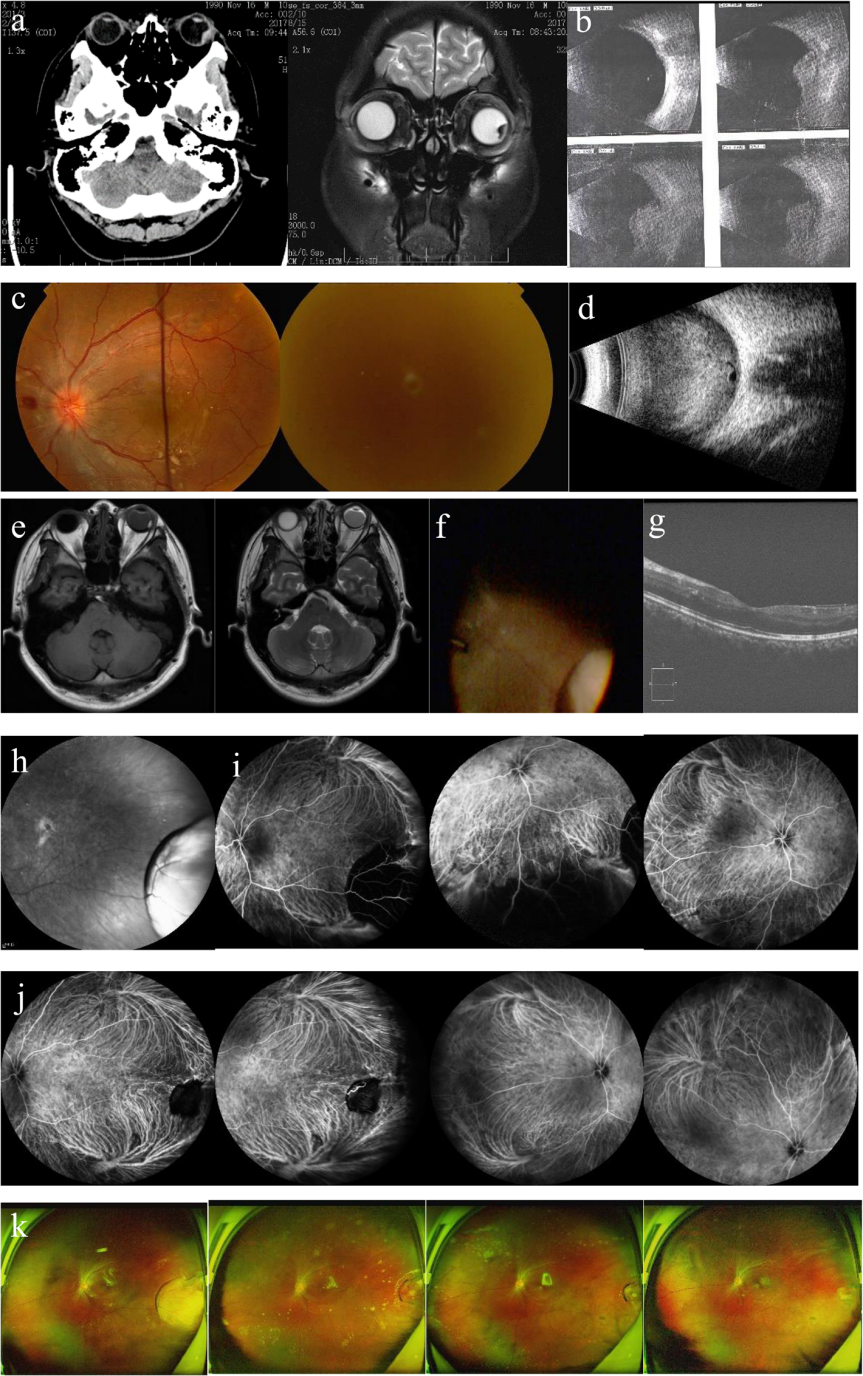
**a** Skull CT and MRI (other hospital). Abnormal signal shadow in the left eyeball (beside the lateral wall). **b** B-ultrasonography (other hospital). A dome-shaped retinal detachment of the left eye. **c** Fundus camera. Disc hemorrhage near the optic disc and yellow and white exudation at posterior pole in the right eye; fundus details could not be evaluated in the left eye because of hemorrhage in the vitreous cavity. **d** B-ultrasonography. A dense, diffuse intravitreal hemorrhage of the left eye. **e** Skull MRI. Abnormal signal shadow in the left eyeball (beside the lateral wall), considering the possibility of intraocular hemorrhage. **f** Intraoperative findings: inferotemporal subretinal focal elevated lesion with clear boundary and yellow color. **g** Three days OCT after the operation. Any obvious abnormality was not observed in the macular of the left eye. **h** Three days of infrared photography after the operation. A dome-shaped bulge in the peripheral part of the inferotemporal region of the left eye. **i** Three days ICGA after the operation. Low fluorescence occlusion and no abnormal fluorescence leakage in the elevated lesion. **j** Half a year ICGA after the operation. The hypofluorescence occlusion area under the retina of the left eye was significantly smaller than before. **k** Scanning laser ophthalmoscopy at 3 days, 3 months, 6 months, and 1.5 years after the operation. Subretinal elevated lesion was gradually reabsorbed of the left eye

A 28-year-old male complained of a sudden loss of vision in the left eye for 1 week when presented to our hospital. He had hypertension for more than 3 years without regular treatment but had no history of any other systemic illness, drug use, myopia, and trauma. Three weeks ago, he was admitted to other hospital due to sudden severe pain and blurred vision of the left eye for 1 h. At the time of admission, the blood pressure was 230/120 mmHg, the intraocular pressure was 69 mmHg, there were conjunctival hyperemia and corneal edema, the pupil diameter was 2.5 mm. Skull CT and MRI (Fig. 1a) detected abnormal signal shadows in the left eye and no abnormalities in the brain. B-ultrasonography (Fig. 1b) indicated an occupying lesion in the left eye. The hospital considered the diagnosis of glaucoma and choroidal occupying lesion. The patient was given symptomatic treatment to reduce the intraocular and blood pressure, including mannitol by intravenous drip, Catinolol Hydrochloride and Brinzolamide eye drops, oral antihypertensives. Due to which, the symptoms improved. However, after 2 weeks, the patient came to our hospital for treatment due to the sudden loss of vision in the left eye. Further examination revealed the following: the blood pressure was 200/120 mmHg, best-corrected visual acuity was 20/20 in the right eye and hand motion in the left eye, and the anterior segment was normal, there were no hyperemia, corneal edema and mydriasis, the intraocular pressure was normal. Fundus camera (Fig. 1c) displayed disc hemorrhage near the optic disc and hard and soft exudates at posterior pole in the right eye; fundus details could not be evaluated in the left eye because of hemorrhage in the vitreous cavity. B-ultrasonography (Fig. 1d) of the left eye revealed a dense, diffuse intravitreal hemorrhage. Skull MRI (Fig. 1e) revealed abnormal signal shadow in the left eyeball (beside the lateral wall), considering the possibility of intraocular hemorrhage. Laboratory examination showed that serum creatinine was 178.0 (53–106 mol/L), and total cholesterol was 6.63 (2.83–5.20 mmol/L). The blood routine and coagulation function were normal, serum infection and immune tests were within normal limits, tumor marker were negative, and no abnormality was found on chest CT or abdominal ultrasound. Thus, the following diagnosis was considered: left eye vitreous hemorrhage, right eye hypertensive retinopathy, hypertension combined with hypertensive crisis, and hyperlipidemia. We recommended a medical consultation for blood pressure reduction, kidney protection, lipid regulation, and other treatments. After the blood pressure was stabilized, vitrectomy combined with silicone oil tamponade of the left eye was performed, retinal vascular abnormalities were ruled out in the vitrectomy performed. In order to keep the refractive medium transparent, silicone oil was used as an endotamponade. The intraoperative finding (Fig. 1f) was an inferotemporal subretinal focal elevated lesion with distinct boundary and yellow color. Since the nature of the elevated lesion could not be determined, it was not treated. No abnormal cells were found in the pathology of vitreous fluid during the operation. Any obvious abnormality was not observed in the macular optical coherence tomography (OCT) (Fig. 1g) of the left eye after surgery. Infrared photography (Fig. 1h) showed a dome-shaped bulge in the peripheral part of the inferotemporal region of the left eye. ICGA (Fig. 1i) showed low fluorescence occlusion and no abnormal fluorescence leakage in the elevated lesion. Combined with the results of systemic and ocular examination, SSRH in the left eye was considered. After close follow-up, the SSRH was found to be gradually reabsorbed, albeit at a slower rate, best-corrected visual acuity was 20/20 in the right eye and 20/25 in the left eye at 1 month after the operation, silicone oil was removed 9 months after surgery. The SSRH was mostly absorbed in his last follow-up visit (1 year after the vitrectomy) (Fig. 1j, k).

## Discussion

SSRH refers to the accumulation of blood between the neurosensory retina and the retinal pigment epithelium which often causes extensive or bullous retinal detachment and is relatively rare in clinical practice. All cases of SSRH reported in the literature have at least one predisposing factor. The systemic risk factors predisposing to SSRH include anticoagulation [2], blood dyscrasia, necrotic tumors, or hypertension [1, 3], while ocular risk factors predisposing to SSRH include macular subretinal neovascularization [4] or retinal vascular anomalies. Kuhli-Hattenbach et al. [5] reported that hypertension increases the pressure in the neovascular lumen among AMD patients receiving anticoagulants or antiplatelet agents, thereby rendering it as a high-risk factor for SSRH. Sosuan et al. [3] reported that hypertension and diabetes mellitus may cause vessel wall changes resulting in SSRH. Although the patient, in this case, is young, the blood pressure is poorly controlled. Except for hypertension, there are no other systemic and ocular risk factors. Herein, we consider that SSRH is associated with high blood pressure. To the best of our knowledge, this is the first report of SSRH in young patients caused by a single factor of hypertension.

When SSRH occurs in the peripheral retina accompanied by vitreous hemorrhage, a precise diagnosis is difficult and is easily misdiagnosed as choroidal melanoma. Therefore, differentiating SSRH from choroidal melanoma is essential. A diagnosis of SSRH and choroidal neoplasm is difficult, and the enucleation caused by misdiagnosis is often reported [6]. The diagnosis should fully combine the clinical manifestations, history, and multimodal imaging of the patient in order to improve the accuracy of clinical diagnosis and reduce the misdiagnosis and mistreatment. Thus, multimodal imaging plays a vital role in the diagnosis of this rare disease.

B-ultrasonography is a sensitive and reliable examination tool that showed flat or hemispherical subretinal solid appearing tumor-like echo combined with retinal detachment with clear boundaries. When combined with vitreous hemorrhage, a large amount of turbidity is observed in the vitreous cavity. B-ultrasonography showed that melanoma cells under the retina absorbed abundant echo energy, and choroidal excavation and shadowing were detected in the orbit formed by internal echoes with smooth attenuation behind a dome-shaped mass lesion or a large mushroom-shaped mass lesion [7]. In the early stage of this case, B-ultrasonography suggested a choroidal occupying lesion in the left eye. The echo of the lesion was masked by vitreous hemorrhage in the later period, and hence, medical history formed the basis of diagnosis.

OCT is a noninvasive, reproducible, and high-resolution technology, which can be used as an auxiliary diagnosis of SSRH. In the early phase, OCT showed that the nerve fiber layer was elevated, and the deep reflexes were weakened [8]. However, in this case, due to the combination of vitreous hemorrhage, OCT scanning was not performed. no abnormality was detected in the OCT scan of the macular area after the operation, and SRH caused by macular lesions was excluded. Nonetheless, due to the location of the lesions, the OCT scan of the bleeding area could not be performed. Thus, the wide-angle OCT proved to be valuable in the diagnosis of SSRH.

ICGA can evaluate the choroidal blood vessels. In SSRH, the elevated lesions showed hypofluorescence shielding, complete shielding choroidal fluorescence, and no abnormal fluorescence leakage. Due to the large amount of tortuous abnormal blood vessels or the destruction of retinal tissues in choroid melanoma, fundus fluorescein angiography (FFA) formed a “double circulation pattern” between the tumor vessels and retinal vessels in the early phase and diffuse or intense fluorescence in the late phase [9]. Previous studies [9] reported that FFA detects blocked fluorescence in the bleeding area during the imaging process. If there is abnormal subretinal neovascularization or retinal macroaneurysm, a dot-like hyperfluorescent spot is observed at the edge or center of hypofluorescence. FFA was not performed because of the poor renal function of this patient.

MRI is helpful in excluding space-occupying lesions, especially in secondary vitreous hemorrhage. It significantly distinguishes SSRH from choroidal melanoma. Thus, MRI examination is critical for the clinical diagnosis of choroidal melanoma. Due to the paramagnetic properties of melanin, it usually shows characteristic signal strength, with high-intensity signal on T1-weighted images and low-intensity signal on T2-weighted images. Due to the different bleeding time, the MRI signal of subretinal hemorrhage lacks certain specificity. T1 and T2 are isointense signals when the bleeding time is < 24 h, and low signals when the bleeding time is 1–3 days. Furthermore, high-intensity signal is detected on T1-weighted images and low on T2-weighted images when the bleeding time is 3–7 days, T1 and T2 are high-intensity signals when the bleeding time is 7–14 days, and T1 is isointense signal and T2 is low-intensity signal when the bleeding time is > 14 days [10]. In this case, both T1 and T2 showed a high-intensity signal, which was consistent with the previous literature, and choroidal melanoma was excluded. The multimodal imaging indicated that this patient was SSRH, and hence, close follow-up was selected as the treatment plan. With the gradual absorption of the subretinal lesion, the diagnosis of SSRH was confirmed.

Previous studies [3] reported that some patients with SRH might initially present with an acute increase in intraocular pressure or acute angle-closure glaucoma. The mechanism of an acute increase in intraocular pressure could be attributed to the massive subretinal hemorrhage that pushed the lens and iris forward, leading to secondary angular-closure glaucoma. Reportedly, massive subretinal hemorrhage causes glaucoma. Since anti-glaucoma drugs are ineffective, evisceration is performed for relieving pain. In this case, the patient presented with an acute increase in intraocular pressure and a decrease in sudden visual acuity at the first visit, leading to the clinical diagnosis of glaucoma. However, MRI and B-ultrasound indicated choroidal mass and excluded the possibility of primary glaucoma.

## Conclusions

This study is a report on the multimodal imaging manifestations of SSRH that are valuable for the diagnosis of this disease. SSRH due to hypertension is rare. A large sample size and long-term follow-up are needed to further understand the etiology, characteristics, and development of the disease.

## Data Availability

All the data supporting the conclusions of this article is included in the present article.
